# Interaction between the Renin–Angiotensin System and Enteric Neurotransmission Contributes to Colonic Dysmotility in the TNBS-Induced Model of Colitis

**DOI:** 10.3390/ijms22094836

**Published:** 2021-05-03

**Authors:** Mariana Ferreira-Duarte, Tiago Rodrigues-Pinto, Teresa Sousa, Miguel A. Faria, Maria Sofia Rocha, Daniela Menezes-Pinto, Marisa Esteves-Monteiro, Fernando Magro, Patrícia Dias-Pereira, Margarida Duarte-Araújo, Manuela Morato

**Affiliations:** 1 Laboratory of Pharmacology, Department of Drug Sciences, Faculty of Pharmacy of University of Porto (FFUP), 4050-313 Porto, Portugal; tiago.pintocfc@gmail.com (T.R.-P.); msrocha@ff.up.pt (M.S.R.); daniela.mennezes@gmail.com (D.M.-P.); mmem89@gmail.com (M.E.-M.); 2 LAQV-REQUIMTE, Faculty of Pharmacy of University of Porto (FFUP), 4050-313 Porto, Portugal; mfaria@ff.up.pt; 3 Unit of Pharmacology and Therapeutics, Department of Biomedicine, Faculty of Medicine, University of Porto, 4200-319 Porto, Portugal; teresa.tsousa@gmail.com (T.S.); fm@med.up.pt (F.M.); 4 Department of Pathology and Molecular Immunology, Institute of Biomedical Sciences Abel Salazar, University of Porto (ICBAS-UP), 4050-313 Porto, Portugal; pdiaspereira@yahoo.com.br; 5 Department of Immuno-Physiology and Pharmacology, Institute of Biomedical Sciences Abel Salazar, University of Porto, 4050-313 Porto, Portugal

**Keywords:** inflammatory bowel disease, IBD, TNBS-induced colitis, colonic dysmotility, angiotensin II, AT_1_ and AT_2_ receptors, nitric oxide, interstitial cells of Cajal, ICC, enteric glial cells, EGC

## Abstract

Angiotensin II (Ang II) regulates colon contraction, acting not only directly on smooth muscle but also indirectly, interfering with myenteric neuromodulation mediated by the activation of AT_1_ /AT_2_ receptors. In this article, we aimed to explore which mediators and cells were involved in Ang II-mediated colonic contraction in the TNBS-induced rat model of colitis. The contractile responses to Ang II were evaluated in distinct regions of the colon of control animals or animals with colitis in the absence and presence of different antagonists/inhibitors. Endogenous levels of Ang II in the colon were assessed by ELISA and the number of AT_1_/AT_2_ receptors by qPCR. Ang II caused AT_1_ receptor-mediated colonic contraction that was markedly decreased along the colons of TNBS-induced rats, consistent with reduced AT_1_ mRNA expression. However, the effect mediated by Ang II is much more intricate, involving (in addition to smooth muscle cells and nerve terminals) ICC and EGC, which communicate by releasing ACh and NO in a complex mechanism that changes colitis, unveiling new therapeutic targets.

## 1. Introduction

Inflammatory bowel disease (IBD) includes Crohn’s disease (CD) and ulcerative colitis (UC). It is a chronic inflammatory condition of the gastrointestinal (GI) tract with prevalence surpassing 0.3% in western countries [1], and it negatively influences the health status and quality of life of patients [2,3], in part due to alterations in colonic transit and reactivity such as the urge to defecate and/or bloody diarrhea [2,3]. Studies on the etiopathology of IBD have mainly focused on immune mechanisms [4,5], and current therapeutic approaches are not always effective in inducing and/or maintaining remission, often depending on type, localization and severity of the disease [6]. Therefore, other pathophysiological mechanisms of IBD should be explored in order to identify new drug targets to improve treatment.

It is nowadays clearly accepted that the renin–angiotensin system (RAS) is involved in inflammation [7], including that of the GI tract [8,9,10,11]. IBD patients who were on angiotensin II (Ang II) type I (AT_1_) receptor antagonists showed less expression of pro-inflammatory cytokines in colonic biopsies than IBD patients who were not on these drugs [8]. Moreover, patients with active CD presented higher levels of Ang II in the intestinal mucosa than healthy controls or patients with UC [12]. Ang II is the main effector peptide of the system and triggers its functions via AT_1_ and AT_2_ receptors [13]. AT_1_ receptors mediate all classic actions of Ang II such as contraction of smooth muscle cells, inflammation, fibrosis, cellular growth and release of aldosterone, while activation of AT_2_ receptors appears to counterbalance those effects [14]. Particularly in the colon, both AT_1_ and AT_2_ receptors were found in the surface epithelium, even though AT_2_ receptors presented a weaker expression [12]. In rats, Ang II induces contractile responses in intestinal muscles via activation of neural AT_1_ receptors and/or AT_1_ receptors located postjunctionally in smooth muscle cells [12,14,15,16]. Although AT_2_ receptors are described in the GI tract, their role in intestinal contraction is only now starting to be reported [17]. The contraction caused by Ang II in the distal colon is lower in experimental IBD than in controls, apparently through recruitment of AT_2_ receptors [17]. Interestingly, it was suggested that enteric neurons participate in Ang II-mediated colonic contraction in dextran sulfate sodium (DSS)-induced colitis in rats [17].

The pathophysiology of IBD is complex in part due to the complex gut network that engages smooth muscle cells, epithelial cells and the intricate enteric nervous system (ENS) to promote gut motility and nutrient absorption [18]. The ENS comprises complex networks of enteric neurons that interact with enteric glial cells (EGC) and interstitial cells of Cajal (ICC) [18,19]. Recently, an involvement of EGC in controlling GI functions has been suggested [18], namely gut motility and secretions [20,21], but ECG are also capable of responding to injury, stress and inflammation [22], producing both pro- and anti-inflammatory responses [23]. In fact, in CD patients [22] and in TNBS-induced ileitis in the guinea pig [24], glial-mediated reactions and EGC mitosis, respectively, were found to have increased. Additionally, ICC are described as the pacemaker cells of the GI tract, regulating spontaneous contractions of the gut [25] and gut motility [26]. In fact, several studies point to a role for ICC in mediating nitrergic [25,27] and purinergic [28] relaxation as well as cholinergic contraction [26] of the GI smooth muscle. In IBD, lower levels of ICC [29] as well as structural changes in these cells have been found [30]. There is almost no information on a putative role of these non-neuronal cells in Ang II-mediated responses, namely colonic contraction.

The aim of our study was to characterize the contractile response to Ang II in three regions of the colon—proximal colon (PC), middle colon (MC) and distal colon (DC)—of rats with 2,4,6-trinitrobenzene sulfonic acid (TNBS)-induced IBD [31] and to look for which cells and mediators participate in that effect of Ang II.

## 2. Results

### 2.1. Assessment of Colitis

**Figure 1:**
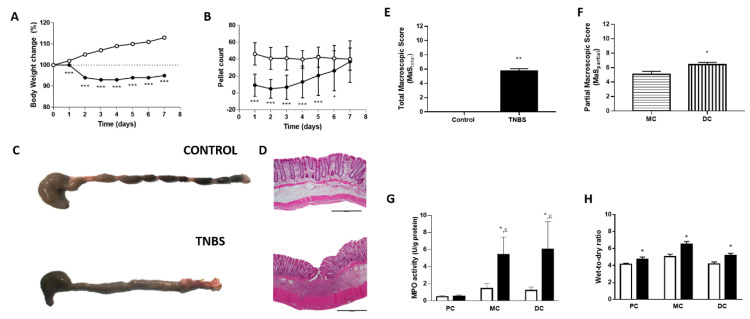
(**A**) Body weight change of control (white circles, *n* = 28) and TNBS-induced rats (black circles, *n* = 87). (**B**) Number of fecal pellets of control (white circles, *n* = 28) and TNBS-induced rats (black circles, *n* = 87). (**C**) Macroscopic and (**D**) microscopic images from the colons of control and TNBS-induced animals. (**E**) Macroscopic score (MaS) of control (white bar, *n* = 28) and TNBS-induced rats (black bar, *n* = 87). (**F**) MaS of the middle colon (MC) and the correspondent distal colon (DC) of TNBS-induced rats (*n* = 87). (**G**) Myeloperoxidase (MPO) activity in the proximal colon (PC), middle colon (MC) and distal colon (DC) of control (white bars; PC: *n* = 12, MD: *n* = 9, DC: *n* = 9) and TNBS-induced rats (black bars; PC: *n* = 16, MD: *n* = 11, DC: *n* = 8). (**H**) Wet-to-dry ratio in the proximal colon (PC), middle colon (MC) and distal colon (DC) of control (white bars; PC: *n* = 29, MC: *n* = 21, DC: *n* = 21) and TNBS-induced rats (black bars; PC: *n* = 20, MC: *n* = 17, DC: *n* = 19). * *p* < 0.05 vs. corresponding controls (except in (D), where * *p* < 0.05 vs. MC), ** *p* < 0.0001, *** *p* < 0.000001 vs. control animals and ^ε^ *p* < 0.05 vs. TNBS-PC.

The body weight and number of pellets were quantified along the time of induction of colitis. From day 1 on, TNBS-induced rats presented lower body weight change than controls (Figure 1A), with a total body weight loss of 5% on day 7. The number of fecal pellets of TNBS-induced animals was also lower than that of controls; this difference was particularly evident at the beginning, but on day 3, the number of fecal pellets from TNBS-induced rats started to increase and was similar to that of controls by the end of the protocol (Figure 1B).

Upon macroscopic evaluation, it was evident that the colons of TNBS-induced rats were filled with pasty stools in their entire lengths and appeared to have a larger diameter than those of control animals, which were filled with ovoid-shaped fecal pellets (Figure 1C). TNBS-induced rats presented a thicker mucosa that usually showed areas of ulceration and hyperemia, particularly in the DC (Figure 1C). Microscopic evaluation of samples from the colon of TNBS-induced rats showed thickening of the colon wall, with several erosive/ulcerative lesions, moderate to extensive inflammatory infiltrate in the mucosa and sub-mucosa and occasional areas of re-epithelization and fibrosis (Figure 1D). TNBS-induced rats had a higher MaS than control animals (Figure 1E). Of interest, in TNBS-induced rats, partial MaS of the DC was higher than that of the MC (Figure 1F).

Colonic inflammation was also confirmed by increased MPO activity in the MC and DC of TNBS-induced animals when compared to the correspondent colonic segment of controls and compared to that of the PC of TNBS-induced animals (Figure 1G). No differences were found between the PC of control and TNBS-induced rats (Figure 1G). In addition, the wet-to-dry ratio (a marker of edema) was higher in all colonic portions of TNBS-induced animals compared with those of control animals (Figure 1H).

### 2.2. Colonic Portions Reactivity to Angiotensin II and Peptide Levels

**Figure 2:**
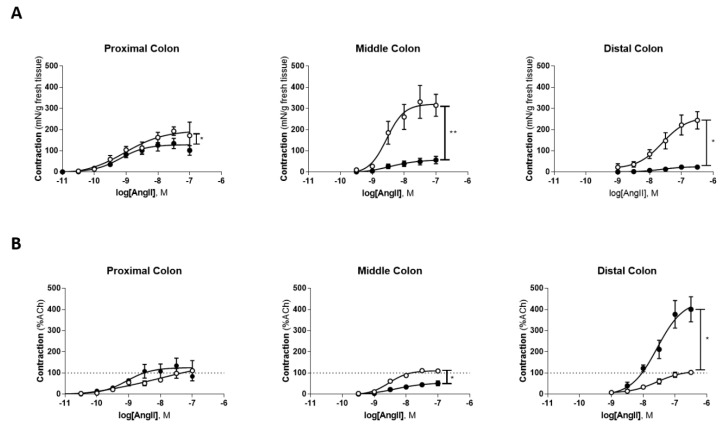
Concentration-response curves to Ang II. (**A**) Ang II-mediated response expressed as tension (mN/g of fresh tissue) in the proximal colon (controls, open circles, *n* = 7 vs. TNBS-induced rats, black circles, *n* = 10), middle colon (controls, open circles, *n* = 5 vs. TNBS-induced rats, black circles, *n* = 10) and distal colon (controls, open circles, *n* = 6 vs. TNBS-induced rats, black circles, *n* = 6); (**B**) Ang II-mediated response expressed as a percentage of ACh (%ACh) in the proximal colon (controls, open circles, *n* = 7 vs. TNBS-induced rats, black circles, *n* = 9), middle colon (controls, open circles, *n* = 5 vs. TNBS-induced rats, black circles, *n* = 10) and distal colon (controls, open circles, *n* = 6 vs. TNBS-induced rats, black circles, *n* = 6). Vertical lines point to statistically significant differences in the E_max_ of the corresponding groups, * *p* < 0.05, ** *p* < 0.0001.

Exogenous administration of Ang II caused a concentration-dependent contraction of the PC, MC and DC in both control and TNBS-induced rats (Figure 2). Interestingly, the contraction promoted by exogenous Ang II was different depending on the colonic region observed and on the units in which data was analyzed.

First, we expressed the contractile response to Ang II as mN of tension *per* g of fresh tissue to have information on the capacity of Ang II to contract a segment of the colon (control and TNBS-induced group). The contraction (mN/g) induced by Ang II was markedly lower in all three colonic portions of TNBS-induced rats than in the correspondent colonic portions of the control group (Figure 2A) (Table 1). Next, we expressed the contractile response to Ang II as a percentage of that induced by ACh 10 µM in the same tissue (%ACh) in order to gather information on the relative capacity of Ang II to contract a segment of the colon when compared to that of another contracturant in the same tissue sample; we selected ACh as the classical contracturant of the colon (ACh). In this case, the contractile response (%ACh) induced by Ang II differed along the colon (Figure 2B). In the PC, there was no difference between control and TNBS-induced animals (Figure 2B) (Table 1), in the MC the response to Ang II was lower in TNBS-induced animals than in controls (Figure 2B) (Table 1) and in the DC Ang II-mediated contraction was markedly higher in TNBS-induced animals than in controls (Figure 2B) (Table 1). Together, these results show that the contractile response to Ang II is decreased in TNBS-induced rats when compared to control rats, but this decrease is not the consequence of a generalized decreased contractile capacity of the inflamed colon. In the PC, that decrease is accompanied by a proportional decrease in the response to Ach, but in the MC, the decrease in Ang II-induced contraction is more marked than that of ACh, while in the DC, the contractile capacity of Ang II is markedly higher than that of ACh (even though Ang II has less force of contraction (mN/g) in TNBS-induced than in control rats). There was no difference in the EC_50_ between groups for the same region of the colon (*p* > 0.05, data not shown).

**Figure 3:**
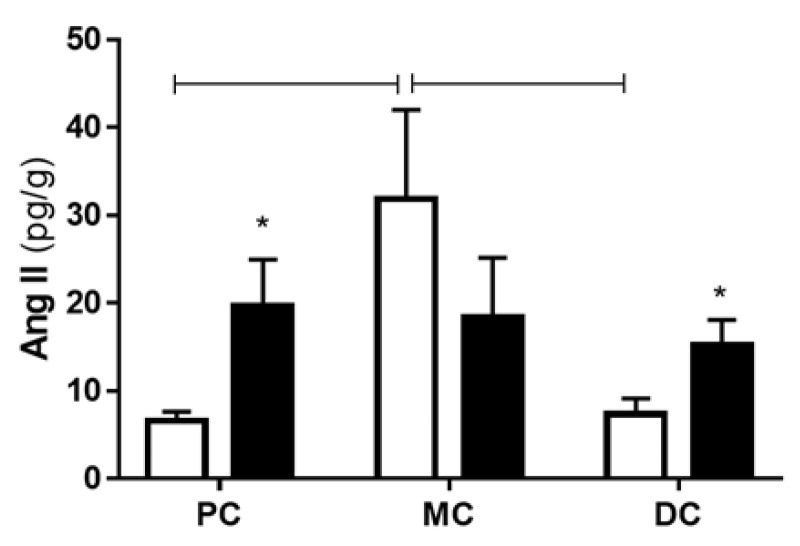
Levels of Ang II of control (white bars) and TNBS-induced rats (black bars) in the PC (controls: *n* = 5; TNBS: *n* = 4), the MC (controls: *n* = 5; TNBS: *n* = 5) and the DC (controls: *n* = 6; TNBS: *n* = 5). * *p* < 0.05 vs. corresponding control; capped lines point to statistically significant differences between groups with *p* < 0.05.

The quantification of the endogenous levels of Ang II in the colonic tissue (PC, MC and DC) of control and TNBS-induced rats showed higher levels of Ang II in the PC and the DC of TNBS-induced animals than in control animals but similar levels between experimental groups in the MC (*p* > 0.05) (Figure 3). Intriguingly, in control rats, the endogenous colonic levels of Ang II were higher in the MC than in the PC and DC (Figure 3). No statistical differences were found between the levels of endogenous Ang II among the colonic portions of TNBS-induced rats (Figure 3).

### 2.3. Role and Expression of Angiotensin II Type I and Type II Receptors

**Figure 4:**
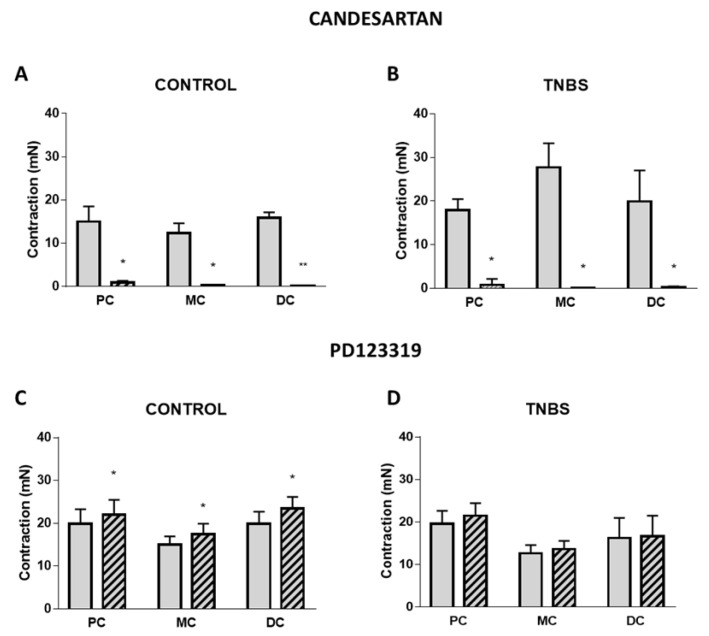
Characterization of the receptors involved in the contraction induced by Ang II in PC, MC and DC. Gray bars and striped bars represent, respectively, the absence and presence of Candesartan 10 nM in (**A**) control (PC: *n* = 6; MC: *n* = 6; DC: *n* = 6) and (**B**) TNBS-induced animals (PC: *n* = 6; MC: *n* = 7; DC: *n* = 7) and the absence and presence of PD123319 100 nM in (**C**) control (PC: *n* = 7; MC: *n* = 6; DC: *n* = 7) and (**D**) TNBS-induced animals (PC: *n* = 11; MC: *n* = 9; DC: *n* = 8). * *p* < 0.05 and ** *p* < 0.0001 vs. Ang II alone.

To further characterize the colonic contractile effect induced by Ang II, the role of AT_1_ and AT_2_ receptors was assessed. Candesartan 10 nM, an AT_1_ receptor antagonist, abolished the contractile effect mediated by Ang II in all three colonic regions of control animals (Figure 4A) and of the TNBS-induced group (Figure 4B), indicating that this was the receptor responsible for the Ang II-mediated contraction. Differently, the presence of the AT_2_ receptor antagonist PD123319 100 nM increased the contractile response to Ang II in the PC, MC and DC of control animals (Figure 4C) but had no effect on the contractile response to Ang II in TNBS-induced rats (*p* > 0.05) (Figure 4D). These results indicate that in control rats, AT_2_ receptors also mediate Ang II-induced contraction but have a contrary effect to AT_1_ receptors (promoting relaxation). In addition, these results show that this AT_2_ receptor-mediated regulation is absent in TNBS-induced rats.

**Figure 5:**
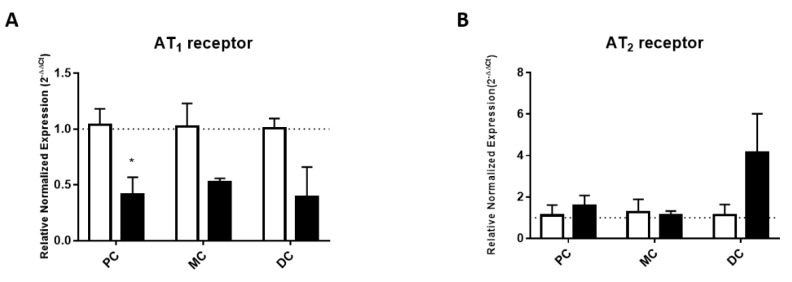
Relative normalized expression (2^−∆∆C^_t_) of (**A**) AT_1_ (PC: *n* = 3, MC: *n* = 2; DC: *n* = 3) and (**B**) AT_2_ (PC: *n* = 2; MC: *n* = 3; DC: *n* = 2) receptors in the controls (white bars) and TNBS-induced (black bars) animals. * *p* < 0.05 vs. corresponding control group.

The expression of the AT_1_ receptor appeared to be decreased in TNBS-induced animals, even though a statistically significant difference was only found in the PC (Figure 5A). Differently, the expression of the AT_2_ receptor was not statistically altered in TNBS-induced rats, although the average values of expression of the AT_2_ receptor in the DC of TNBS-induced rats were numerically higher (Figure 5B). Overall, these results point to decreased expression of the AT_1_ receptor but similar expression of the AT_2_ receptor in the colons of TNBS-induced rats when compared with controls.

### 2.4. Neural and Non-Neural Cells Mediating Angiotensin II-Induced Colonic Contraction

**Figure 6:**
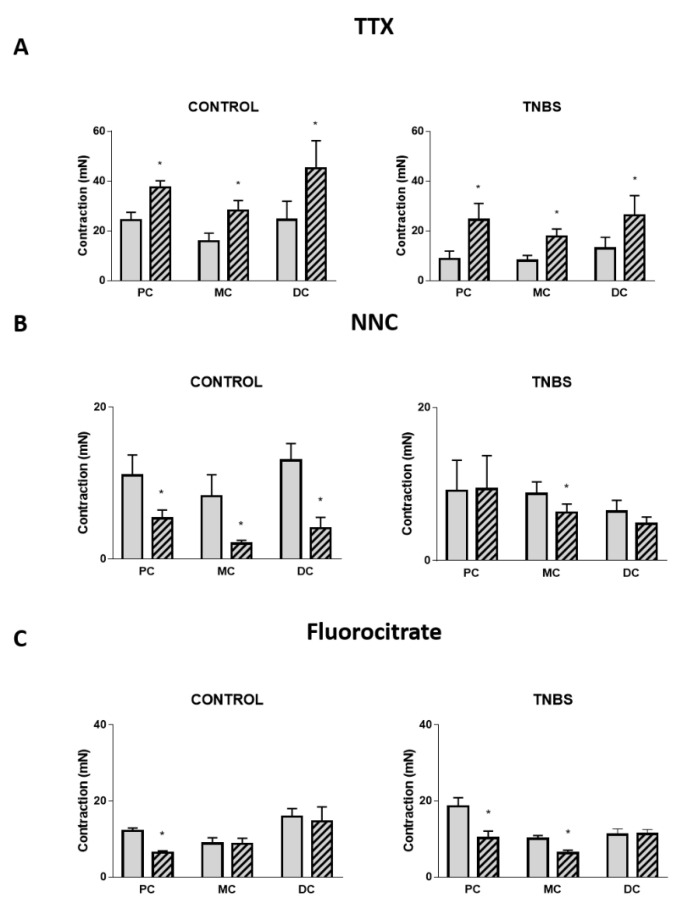
Contractile response to Ang II in the PC, MC and DC in the absence (gray bars) and presence (striped bars) of (**A**) TTX 1 µM in control (PC: *n* = 6; MC: *n* = 5; DC: *n* = 5) and TNBS-induced animals (PC: *n* = 6; MC: *n* = 4; DC: *n* = 6); (**B**) NNC 55-0396 10 µM (PC) or 30 µM (MC and DC) in control (PC: *n* = 8; MC: *n* = 8; DC: *n* = 6) and TNBS-induced animals (PC: *n* = 6; MC: *n* = 5; DC: *n* = 6); and (**C**) fluorocitrate 300 µM in control (PC: *n* = 7; MC: *n* = 5; DC: *n* = 5) and TNBS-induced animals (PC: *n* = 5; MC: *n* = 6; DC: *n* = 7). * *p* < 0.05 vs. Ang II alone.

Because the colonic tissue is a complex network involving different types of cells, we further evaluated the response to different antagonists and blockers. Tetrodotoxin (TTX 1 µM), a neurotoxin that blocks Na^+^ voltage-gated ion channels impairing neurotransmitter release from myenteric neurons, significantly increased the contractile response to Ang II in all colonic regions of both control (Figure 6A left) and TNBS-induced rats (Figure 6A right). Further analysis revealed a statistically significant difference between control and TNBS-induced groups (*p* < 0.05) but not between tissues nor an interaction between the two factors (*p* > 0.05).

NNC 55-0396 (a calcium T-type channel blocker, 10 µM or 30 µM) was used as an ICC inhibitor. This drug decreased Ang II-induced contraction in all colonic portions of control animals (Figure 6B left). However, in TNBS-induced animals, this effect was limited to the MC (Figure 6B right). Further analysis revealed a statistically significant difference between control and TNBS-induced groups (*p* < 0.05) but not between tissues nor an interaction between the two factors (*p* > 0.05).

Furthermore, in control animals, fluorocitrate (FC, 300 µM), a glial cells’ inhibitor, decreased the contractile response to Ang II in the PC but had no effect in the other portions of the colon (Figure 6C left). In TNBS-induced animals, this effect was visible not only in the PC, but also in the MC (Figure 6C right). Further analysis revealed a statistically significant difference between colonic regions (*p* < 0.05) but not between groups nor an interaction between the two factors (*p* > 0.05).

Overall, these results indicate that Ang II-induced colonic contraction is under the regulation of both neuronal (inhibitory) and non-neuronal cells (excitatory), and that the non-neuronal component is altered in TNBS-induced colitis.

### 2.5. Mediators Involved in Angiotensin II-Induced Colonic Contraction

**Figure 7:**
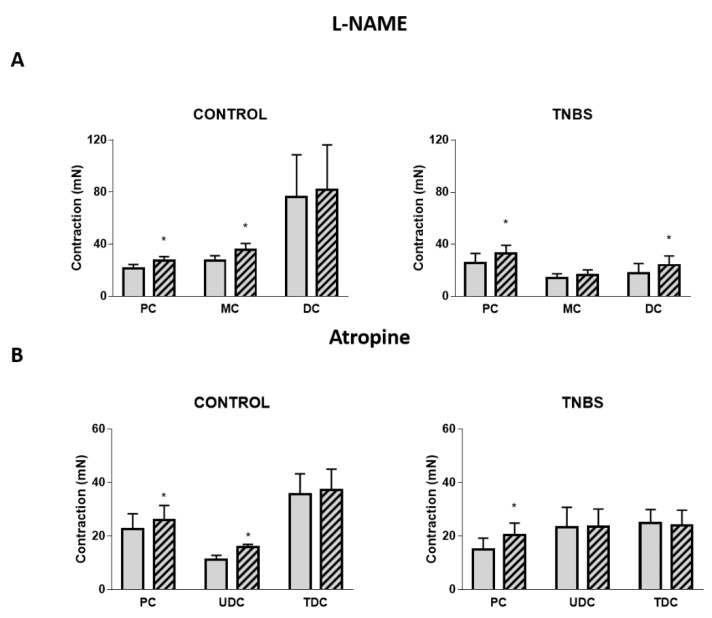
Contractile response to Ang II in the PC, MC and DC in the absence (gray bars) and presence (striped bars) of (**A**) L-NAME 100 µM in control (PC: *n* = 5; MC: *n* = 6; DC: *n* = 6) and TNBS-induced animals (PC: *n* = 6; MC: *n* = 5; DC: *n* = 6); and (**B**) atropine 1 µM in control (PC: *n* = 5; MC: *n* = 6; DC: *n* = 6) and TNBS-induced animals (PC: *n* = 6; MC: *n* = 5; DC: *n* = 5). * *p* < 0.05 vs. Ang II alone.

We also assessed the role of several mediators in Ang II-mediated contraction of the colon. A nitric oxide synthase (NOS) inhibitor (L-NAME, 100µM) increased the contractile response to Ang II in the PC and MC of control rats (Figure 7A left) and in the PC and DC of TNBS-induced rats (Figure 7A right). Atropine, a non-selective muscarinic antagonist, increased the contractile response to Ang II in the PC and MC of control rats (Figure 7B left) and in the PC of TNBS-induced rats (Figure 7B right). Phentolamine increased the contractile response to Ang II in the DC of control rats (17.86 ± 8.40 mN vs. 20.38 ± 8.50 mN, Ang II alone vs. Ang II + phentolamine, respectively; *p* < 0.05, n = 5; graphs not shown). Propranolol, caffeine or suramine did not alter the response to Ang II, either in control or TNBS-induced rats, in any of the three colonic portions analyzed (*p* > 0.05; data not shown).

**Figure 8:**
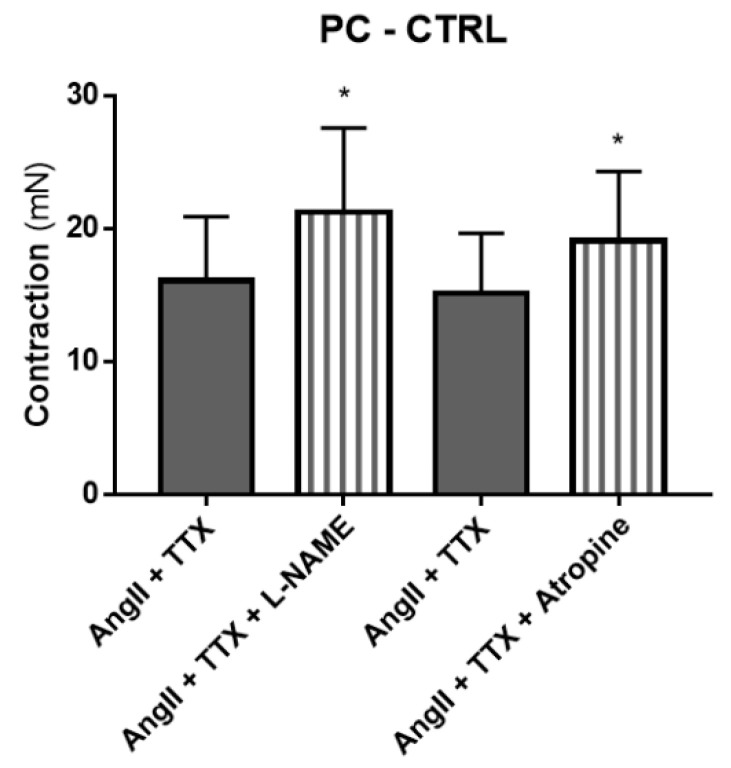
Contractile response to Ang II in the PC of control animals in the presence of TTX 1 µM (dark gray bars) compared to the presence of TTX and L-NAME 100 µM (*n* = 6) or atropine 1 µM (*n* = 5) (striped bars). * *p* < 0.05 vs. Ang II with TTX.

Since the results with L-NAME, atropine and phentolamine exhibited the same effects as TTX on Ang II-induced contraction (increased), we explored the possibility of the correspondent mediators (NO, muscarinic receptors and α-adrenoceptors, respectively) being dependent on prejunctional neuronal activation (evidenced by the effect of TTX). Co-administration of TTX and L-NAME did not alter the colonic contractile response to Ang II vs. TTX alone in the MC of control animals or in the PC and DC of TNBS-induced rats (*p* > 0.05; data not shown). Similarly, co-administration of TTX and atropine did not alter the colonic contractile response to Ang II vs. TTX alone in the MC of control animals or in the PC of TNBS-induced rats (*p* > 0.05; data not shown). The response to Ang II was also not altered when the PC of control rats was incubated with TTX or with TTX plus phentolamine (*p* > 0.05; data not shown). However, co-administration of TTX and L-NAME and of TTX and atropine further increased the colonic contractile response to Ang II vs. TTX alone in the PC of control animals (Figure 8). Overall, these results suggest that in the PC of control rats, NO and muscarinic receptors participate in Ang II-induced contraction (decrease it) but not as a part of a mechanism involving neurons. Differently, the role for NO, muscarinic receptors and α-adrenoceptors in mediating Ang II-induced contraction in other colonic regions of control and TNBS-induced rats is dependent on the activation of enteric nerve terminals.

## 3. Discussion

**Figure 9:**
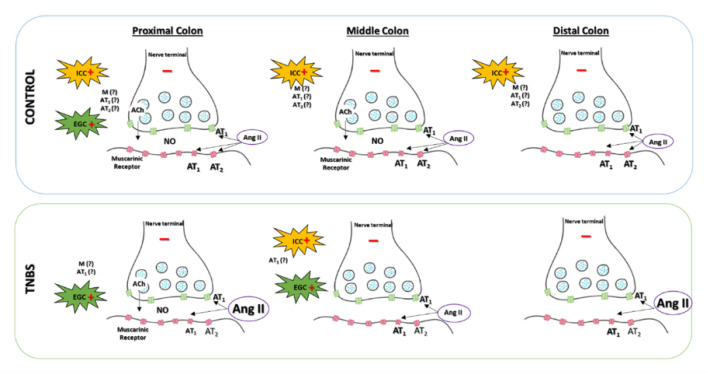
Schematic representation of the effect of Ang II in colonic reactivity along the colon of control (**top panel**) and TNBS-induced rats (**bottom panel**). Ang II activates AT_1_ receptors, contracting all colonic regions of control and TNBS-induced rats. Ang II also activates AT_2_ receptors along the colon of control animals, promoting colonic relaxation, but this effect is not observed in TNBS-induced rats. Animals with TNBS-induced colitis show higher tissue concentration of endogenous Ang II and decreased expression of AT_1_ receptors but similar expression of AT_2_ receptors. The intricate, Ang II-mediated colonic contraction involves a neuronal inhibitory component and a non-neuronal excitatory component. The neuronal component involves activation of muscarinic receptors and production of NO. The non-neuronal component includes activation of EGC and/or ICC, dependent on the colonic region. EGC—enteric glial cells; ICC—interstitial cells of Cajal; M—muscarinic receptor; NO—nitric oxide; (+)—excitatory mechanism; (−)—inhibitory mechanism.

Our study shows for the first time that TNBS-induced colitis is associated with decreased Ang II-mediated contraction along the entire rat colon. We also report evidence that Ang II-mediated contraction results from an intricate network between smooth muscle cells, nerve terminals, ICC and EGC, communicating through muscarinic receptors and NO release. Furthermore, we show that this complex mechanism is altered in rat TNBS-induced colitis (Figure 9).

IBD can affect scattered parts of the colon in Crohn’s disease or continuously, with a distal-to-proximal pattern, in ulcerative colitis. Our study advances the state-of-the-art and pursues translational relevance in colitis because instead of studying the colon as a uniform target organ of IBD, we studied: (1) the PC—no macroscopic alterations, similar MPO activity but increased wet-to-dry ratio vs. controls; (2) the MC—evident macroscopic alterations and increased MPO activity and wet-to-dry ratio; and (3) the DC—increased MPO activity and wet-to-dry ratio than in controls but more marked MaS than the MC. With this approach, our study is the first to report a generalized decrease in the response to Ang II along the colon of rats with TNBS-induced colitis when compared to control rats. However, because we looked for the absolute and relative forces of contraction induced by Ang II, we could also report interesting differences in this decreased response to Ang II. Indeed, we report that minimum inflammatory alterations (PC) equally affect Ang II and ACh contractile function, while evident inflammatory damage (MC) further decreases the relative contractile effect of exogenous Ang II over that of ACh. However, marked inflammatory damage (DC) seems to favor the contractile response of Ang II over that of ACh, which might represent a compensatory response to the loss of function caused by extensive inflammatory injury (Figure 2). Inflammatory damage can nonspecifically affect the contractile capacity of the colon by decreasing the ability of the smooth muscle to contract, either by direct lesion of smooth muscle cells or by steric disturbance of the contractile mechanics across the colonic wall [32]. However, it can also disturb the interplay between the cells/receptors/mediators operating in the colon wall [32]. 

The lower reactivity to Ang II might be the result of an imbalance in function and/or expression of Ang II receptors [33]. Our functional results with AT_1_ (candesartan) and AT_2_ (PD123319) antagonists showed that along the colon of control rats, Ang II-mediated contraction resulted from activation of AT_1_ receptors, with a regulatory relaxation mediated by the AT_2_ receptor that is blunted in TNBS-induced rats (Figure 4). This would anticipate an increase in Ang II-induced contraction in TNBS-induced rats, which would be in clear contrast with our findings. However, we observed higher tissue levels of Ang II (Figure 3), also reported in IBD patients [34,35], and these could contribute to downregulation of AT_1_ receptor mRNA expression (as we observed), even without altering the expression of AT_2_ receptors (Figure 5). This could justify the lower reactivity to Ang II observed in TNBS-induced rats when compared to controls. Although a contractile role for the AT_1_ receptors has already been described in the healthy colon [15,36] and in experimental colitis [17], Zizzo and co-workers reported decreased Ang-II mediated contraction (% carbachol, an ACh mimetic) in the distal colon of DNBS-induced rats, associated with increased expression of both AT_1_ and AT_2_ receptors [17]. DNBS-induced colitis is a chemically-induced model of colitis very similar to that of TNBS, and experiments were also carried out after 1 week [17]. However, we used TNBS-induced animals that presented mild or moderate colitis [37], but the animals used by Zizzo et al. might have had more severe colitis, considering the reported MaS and MPO activity levels [17]. Furthermore, we report that two colonic segments just 2.5 cm apart (which we named MC and DC) present different relative responses to Ang II, while the authors mentioned above did not specify the exact location of the colon portion used. It is also possible that the downregulation of AT_1_ receptors observed in our study serves as a mechanism to control TNBS-induced inflammatory damage because knock-out mice for AT_1_ receptors have been shown to develop less severe colitis than wild-type mice [38,39]. 

Our results with TTX, NNC 55-0396 and FC support the view that the contractile effect of Ang II results from direct postjunctional activation of Ang II receptors in smooth muscle cells and also from indirect modulation of prejunctional release of neurotransmitters from nerve terminals, ICC and EGC [40,41,42,43,44,45,46]. Further, we distinguished not only a neuronal inhibitory regulation but also a non-neuronal (ICC, EGC) excitatory regulation, which is altered in TNBS-induced colitis. Results with TTX support the view of an indirect inhibitory neuronal mechanism triggered by exogenous Ang II in all three regions of the colon of both control and TNBS-induced animals (Figure 6A). The study of Zizzo and collaborators also described an inhibitory neuronal pathway activated by Ang II in control animals but reported that it was blunted in DNBS-induced rats [17]. Other studies indicate that Ang II activates an excitatory neuronal mechanism in the mouse colon [15] and human sigmoid colon [36]. Non-neural cells such as ICC and EGC are also a major component of enteric neuromodulation [47] and play a role in the complex network that ensures proper colonic contraction [20,25,26]. To our knowledge, our study is the first to explore the putative role of ICC and EGC in Ang II-mediated contraction of the rat colon. Our results with NNC 55-0396 showed that in the three colonic regions of control rats, ICC contributed positively to Ang II-induced contraction, whereas for TNBS-induced animals, this mechanism was observed only in the MC (Figure 6B). Several studies have shown that ICC are lost or damaged in many inflammatory disorders, being decreased in the colons of patients with UC (inflamed regions compared to non-inflamed regions) [29] and in the rat TNBS model of ileitis [46] and colitis [48]. A role for ICC in synthesizing NO has also been suggested in experimental colitis [49]. Concerning EGC, our results show that they contribute to Ang II-induced contraction in the PC of control rats and in the PC and MC of TNBS-induced animals (Figure 6C), suggesting that during inflammation, Ang II activates EGC. Intriguingly, we found no effect of FC in the DC of TNBS-induced animals, which was the region presenting the most severe inflammatory damage, possibly resulting in loss of EGC [29]. Of interest, in a TNBS-induced model of ileitis in rats, inflammation stimulated EGC proliferation that was responsible for the maintenance of ACh release from enteric neurons [50]. Generally, TNBS-induced inflammation disturbs this intricate enteric network by altering the regulatory effect of non-neuronal cells: The regulatory role of the ICC seems to be lost, while that of the EGC appears to be recruited to support Ang II-induced contraction in a proximal-to-distal direction. Interestingly, it is reported that inflammation activates EGC, and that this could be associated with either a pro-inflammatory state or an attempt to resolve inflammation [22,23].

ACh and NO are the main neurotransmitters of the ENS, mediating contraction [51] and relaxation [52,53], respectively. Surprisingly, experiments with atropine revealed that activation of muscarinic receptors counteracted Ang II-mediated contraction in the PC and MC of control rats and in the PC and DC of TNBS-induced rats (Figure 7B), which is in contrast with previously reported data [15]. In vascular tissues, a role for direct M_3_ receptor-mediated relaxation of the smooth muscle has been described [54,55], but in the colon, activation of M_3_ receptors by ACh mediates contraction [56]. Even considering that Ang II decreases the release of ACh from nerve terminals [57], the effect of atropine should be a decrease in Ang II-mediated contraction. As such, these results further strengthen the view of an indirect pathway mediating Ang II contraction. Interestingly, our results with L-NAME showed a similar pattern to those with atropine (Figure 7A). ACh stimulates the production and release of NO from nerve terminals in the proximal [58,59] and distal [58] colon, but not in the middle colon [58,59]. Furthermore, ACh and NO might also act as cotransmitters in some enteric nerves [60] and exert neuromodulatory actions between them [61,62,63]. Our double antagonist protocol showed that in the PC of control rats, the inhibitory pathway involving both muscarinic receptors and NO is not (or not only) occurring through prejunctional nerves (Figure 8). Differently, in the other colonic regions, that inhibitory pathway seems to be entirely dependent on nerve terminals. Alterations in nitrergic neurotransmission occur in acute experimental colitis [53,64]. Zizzo et al. have previously suggested an inhibitory role for NO in modulating Ang II-induced colonic contraction in the distal colon of DNBS-treated rats [17], and it was reported that NO release from neurons or enteric glia is stimulated by the activation of nicotinic receptors in animals with experimental colitis but by muscarinic receptors in controls [64]. In an *Escherichia coli* endotoxin-induced model of colitis, expression of iNOS was found to be increased in the inflamed colonic tissue, and its inhibition led to an increase in the contractile response to ACh, showing once again an interaction between the two molecules [65].

In conclusion, our study showed that in rats with mild/moderate experimental colitis caused by TNBS, Ang II-induced contraction was decreased. This possibly resulted from downregulation of AT_1_ receptors and alterations in the communication between the intricate network of neuronal and non-neuronal cells (as ICC and EGC) by NO and muscarinic receptors.

## 4. Materials and Methods

### 4.1. Animals

Male Wistar Han rats were raised and maintained in the rodent animal facility of ICBAS-UP and housed 2–3 per cage in Rat IVC cages with controlled ventilation and a stable temperature (20–24 °C) and relative humidity (40–70%) under a regular 12 h light/dark cycle. All animals had *ad libitum* access to a laboratory rodent diet and tap autoclaved water. Enrichment materials like nesting paper and play tunnels were provided.

### 4.2. Colitis Induction

TNBS-induced colitis is a well-established animal model of IBD, widely used because it is simple to implement and cost-effective [31]. This model was introduced by Morris et al. [66], but several variations of the original protocol have been introduced [31,37]. Briefly, to achieve TNBS-induced colitis, an ethanolic TNBS solution should be instilled in the rectum. In this model, the alcohol is essential for disrupting the intestinal epithelial cell barrier, enabling TNBS to penetrate the bowel wall and resulting in haptenization of intestinal proteins, which signalizes them as targets of the host immune system [67]. 

Male Wistar Han rats of 8 to 12 weeks of age (littermates) were randomly assigned to the control or TNBS group. Control animals were not subjected to any manipulation. On day −1, rats of the TNBS group were individually housed in clean cages and fasted overnight with ad libitum access to a 5% sucrose solution. Then, on day 0, they were anesthetized ad effectum with isoflurane (Isoflo®, Esteve, Barcelona, Spain) and a 21% ethanolic solution of TNBS (Sigma-Aldrich Inc, St. Louis, MO, USA) (20 mg/rat; 250 µL) was rectally instilled (adapted from Morris et al. [66], using a 7.6 cm ball-tipped catheter (ST1 75-0285, Harvard Apparatus)). Rats were maintained in a head-down position for 60 s to ensure TNBS distribution and no leakage. Analgesia was provided, with daily administration of paracetamol (Paracetamol®, Farmoz, PO, 500 mg/kg) in a honey-based solution from day 0 until day 7. Tramadol (Labesfal, 100 mg/2 mL, SC) was only administered when animals presented severe signs of discomfort. In addition, metoclopramide (Labesfal, 1 mg/kg, SC) was administered from day −1 (before induction) to day 2 of the protocol to assure intestinal motility. All animals were monitored daily throughout the protocol to ensure their well-being and to record individual body weight and the number of fecal pellets. No mortality was observed in this study.

### 4.3. Evaluation of Macroscopic and Microscopic Inflammatory Damage

On day 7 or 8, animals were sacrificed by decapitation (Small Decapitator, Harvard Apparatus). The abdomen was opened, and the general appearance of the colon and surrounding tissues was observed. Next, the colon was excised and gently cleaned of fecal content using Krebs–Henseleit solution ((in mM): 118 NaCl, 4.8 KCl, 2.5 CaCl_2_·2H_2_O, 1.2 NaH_2_PO_4_·H_2_O, 1.2 MgSO_4_·7H_2_O, 25 NaHCO_3_, 0.02 Na_2_EDTA, 0.3 Ascorbic Acid and 11 glucose mono-hydrated). Following that, four intact, 1 cm-long segments were cut as previously reported [37] and used in subsequent in vitro functional experiments (see below): two segments from the proximal colon (PC), 1 cm distal to the cecum; one segment from the middle colon (MC), 4.5 cm above the anus; and another segment from the distal colon (DC), 2 cm above the anus. These 3 areas were chosen to represent an area without obvious lesions (PC), an area upstream of the visible area of injury (MC) and another area close to the obvious area of injury (DC).

Subsequently, the rest of the colon was opened longitudinally to allow macroscopic evaluation of mucosal TNBS damage and attribution of a macroscopic score (MaS), as previously described [37] (Table 2). MaS was calculated as the mean between partial MaS of MC and DC because PC was the region without obvious macroscopic alterations. MaS assessment allowed categorization of experimental colitis in mild, moderate or severe colitis [37]. Only animals that presented mild or moderate colitis were used in this study.

Microscopic inflammatory damage was evaluated in a 1.5 cm-long, full-thickness segment of the colon, as previously described [37]. For that, the segment was fixed in 10% buffered formalin, embedded in paraffin wax and cut in 3 µm, full-thickness sections that were stained with hematoxylin and eosin.

### 4.4. Myeloperoxidase Assay

Myeloperoxidase (MPO) activity, a marker for neutrophil inflammation, was assessed as reported by Dinis-Oliveira et al. [68]. Briefly, tissues from three colonic regions were excised, pat-dried with gauze, weighted and homogenized on ice in ice-cold, 50 mM phosphate buffer (1:4 *m/v*) with 0.1% (*v/v*) Triton X-100, pH 7.4, followed by centrifugation at 15.700 g for 10 min at 4 °C. Supernatants were stored at −80 °C until use. Protein quantification was performed according to Lowry et al [69], using bovine serum albumin as a standard. 

For the MPO assay, supernatants were submitted to three cycles of snap freezing (−80 °C to room temperature). In 96-well plates, to each well was added 50 μL of supernatant and 50 μL of TMB (final concentration 7.5 mM) dissolved in dimethyl sulfoxide. Immediately before plate reading, 50 μL of H_2_O_2_ (final concentration 1.5 mM) dissolved in phosphate buffer (Na_2_HPO_4_·2H_2_O 50 mM, pH 5.4) was added to each well. MPO activity was monitored by recording the absorbance increase at 655 nm at 37 °C for 3 min. One enzyme unit (U) was defined as the amount of enzyme that utilized 1 μmol of H_2_O_2_ per minute as described by Marquez and Dunford [70]. Results are expressed in U/g of protein (ε = 3.9 × 10 ^4^ M^−1^ cm^−1^).

### 4.5. Characterization of the Reactivity of Colonic Tissue to Angiotensin II

Intact, 1 cm-long segments of the colon of control and TNBS-induced rats were mounted in isolated organ baths containing Krebs–Henseleit solution, continuously aerated (95% O_2_ + 5% CO_2_) and kept at a constant temperature of 37 °C. Segments were subjected to an initial resting tension of 1 g and left to equilibrate for at least 20 min until spontaneous mechanical activity was observed. Next, all segments were challenged with acetylcholine (ACh, Sigma-Aldrich, St. Louis, MO, USA) 10 µM twice to confirm the viability and stabilization of the tissue. After that, increasing concentrations of Ang II (Sigma-Aldrich, St. Louis, MO, USA) were added to the baths in a non-cumulative manner (PC: 100 pM–30 nM; MC: 300 pM–100 nM; DC: 1 nM–300 nM) for approximately 2 min. To prevent tachyphylaxis, there were intervals of 1 h between the addition of different concentrations of Ang II.

Next, we performed a second set of experiments in order to characterize the receptor(s) mediating the contractile response to Ang II. For that, the contractile response to submaximal concentrations of Ang II (PC: 10 nM; MC: 30 nM; DC: 100 nM) was evaluated in the absence and presence of candesartan 10 nM (dissolved in physiological saline; a kind gift from Dr. Fredrik Palm, Uppsala University, Sweden), an AT_1_ receptor antagonist, or of PD123319 100 nM (Sigma-Aldrich, St. Louis, MO, USA), an AT_2_ receptor antagonist. The antagonists were left in contact with the tissue for 20 min before the second response to Ang II was obtained.

After that, we wanted to evaluate which cells of the enteric nervous system were involved in the contractile response to Ang II. For that, we used the same protocol as described above but using drugs that inhibited the function of nerve terminals, EGC and ICC. Thus, the contractile response to the same concentrations of Ang II indicated above were tested in the absence and presence of tetrodotoxin (TTX) 1 µM (Sigma-Aldrich, St. Louis, MO, USA), fluorocitrate (FC) 100 µM (Sigma-Aldrich, St. Louis, MO, USA) or (1S,2S)-2-(2-(N-[(3-Benzimidazol-2-yl)propyl]-N-methylamino)ethyl)-6-fluoro-1,2,3,4-tetrahydro-1-isopropyl-2-naphtyl cyclopropanecarboxylate dihydrochloride hydrate (NNC 55-0396) (PC: 10 µM; MC and DC: 30 µM) (Sigma-Aldrich, St. Louis, MO, USA). TTX was used as a neurotoxin because it inhibits Na^+^ voltage-gated ion channels, thus inhibiting the firing of action potentials in neurons [71]. FC is a metabolic toxin that selectively inhibits the tricarboxylic acid cycle in glial cells by blocking the enzyme aconitase [72]. NNC 55-0396 was used as a pharmacological tool to inhibit the activity of the ICC because it selectively blocks T-type, high-voltage-activated Ca^2+^ channels and does not inhibit other ion channels [73].

Furthermore, we repeated the same protocol with different drugs to evaluate the mediators involved in colonic contraction in response to Ang II. We investigated the role of NO, a potent vasodilator, by decreasing its production with NG-nitro-L-arginine methyl ester (L-NAME, 100 µM, Sigma-Aldrich, St. Louis, MO, USA), an inhibitor of nitric oxide synthase (NOS). In addition, we tested some antagonists to investigate the role of the correspondent receptors, namely atropine 1 µM (Sigma-Aldrich, USA) for muscarinic receptors, caffeine 300 µM (Sigma-Aldrich, St. Louis, MO, USA) for adenosine receptors, suramine 100 µM (Sigma-Aldrich, St. Louis, MO, USA) for P2-purinoceptors, phentolamine 3 µM (Sigma-Aldrich, St. Louis, MO, USA) for α-adrenergic receptors and propranolol 1 µM (Sigma-Aldrich, St. Louis, MO, USA) for β-adrenergic receptors.

Finally, we performed a third set of experiments in order to evaluate which cell was responsible for the release of the enteric mediators. In this experimental protocol, colonic segments were first incubated with TTX 1 µM for 30 min, and the response to Ang II (PC: 10 nM; MC: 30 nM; DC: 100 nM) was recorded. Afterward, tissues were washed for 1 h, every 10 min, and then co-incubated for 30 min with TTX 1 µM plus L-NAME 100 µM, atropine 1 µM or phentolamine 3 µM, which were added to the organ bath 10 min after TTX 1 µM (20 min incubation time). After this double incubation, colonic segments were again challenged with Ang II (PC: 10 nM; MC: 30 nM; DC: 100 nM).

At the end of each experimental protocol, tissues were dried on filter paper and weighed at the end of the experimental protocol, left overnight at room temperature and then weighed again in order to quantify the wet-to-dry ratio. 

### 4.6. Extraction and Quantification of Angiotensin II Levels in Colonic Samples from PC, MC and DC

Immediately after the euthanasia of some animals (control and TNBS-induced), colonic portions (PC, MC and DC from control and TNBS-induced rats) weighting about 150–250 mg were collected. Tissues were homogenized with 5 mL homogenization buffer (sodium phosphate 0.1 M, sucrose 0.34 M and NaCl 0.3 M) with a protease inhibitor cocktail (Roche Complete mini #11836153001—one pill dissolved in 1 mL of distilled water; 100 µL of this solution was added to the homogenization buffer). Next, homogenates were centrifuged at 10000 rpm for 10 min at 4 °C, and supernatants were immediately separated to a different vial and kept frozen at −80 °C until further analysis.

Ang II was extracted by solid phase extraction (SPE) (Discovery® DSC-Ph SPE Tube, Supelco, PA, USA). SPE columns were activated using solvents in the following order: methanol, tetrahydrofuran, hexane, methanol and ultrapure water. Next, samples were applied to each column and eluted with ultrapure water. The aqueous extract was kept at −20 °C for re-extractions, if needed. Following that, ultrapure water followed by a solution of acetic acid 4% were passed through the columns and discharged. Finally, a mixture consisting of ethanol:acetic acid:water (90:4:6) (this mixture should be prepared on time from cold stock solutions) was used to elute Ang II. Samples were concentrated by evaporation with nitrogen and then lyophilized and kept frozen at −80 °C until needed.

Quantification of endogenous tissue Ang II levels in the samples from PC, MC and DC (control and TNBS-induced rats) was performed using a peptide enzyme immunoassay (EIA) (Peninsula Laboratories International, Inc., San Carlos, CA, USA), following manufacturers’ instructions (Protocol II). Results are presented as pg of Ang II per g of colonic portion (pg/g).

### 4.7. Expression of Angiotensin II Type I and II Receptors in Colonic Samples from PC, MC and DC by qPCR

Prior to sampling for RNA, colonic portions (PC, MC and DC of control and TNBS-induced rats) were excised and collected in a tube containing RNAlater® and kept at −20 °C until analysis. Total RNA was then extracted from 29.17–194.00 mg colonic tissue using PurezolTM RNA Isolation Reagent (Biorad, Hercules, CA, USA), and reverse transcription was performed from 1 µg RNA using the Xpert cDNA Synthesis Kit (GRiSP Research Solutions, Porto, Portugal) according to the manufacturer’s instructions. RNA quantification and purity were measured spectrophotometrically at 260/280 in a plate reader (BioTek Synergy) adapted for 2 µL volume readings (Take3 adapter, BioTek, Winooski, VT, USA). Real-time polymerase chain reactions (qPCRs) were carried out applying SsoAdvanced™ Universal SYBR® Green Supermix (BioRad, Hercules, CA, USA) and run on a Bio-Rad CFX 96 thermalcycler using rat-specific primer pairs for the receptors AT_1_ and AT_2_, shown in Table 3 [74]. Data was normalized to the expression of housekeeping genes Papbn, Hprt-1 and Hmbs, whose expression was altered by sex, using the delta-delta C_t_ (**ΔΔ**C_t_) method [75]. Every biological replicate was run in three qPCR technical triplicates. The thermal cycling protocol was the following: polymerase activation, 30 s at 98 °C, then denaturation for 15 s at 95 °C, followed by annealing/extension and plate read, 30 s at 60 °C, for 40 cycles.

### 4.8. Data Collection and Statistical Analysis

For each experiment on the response to Ang II in each colonic segment, a concentration–response curve for Ang II was generated by best fit nonlinear regression (variable slope) using GraphPad Prism 7 software (La Jolla, CA, USA), and the correspondent Emax (maximum contractile effect induced by Ang II) and EC_50_ (concentration of Ang II that caused 50% of the maximum response) were obtained. Average concentration–response curves were generated for the response to Ang II obtained in tissues from control and TNBS-induced rats. 

All data are mean ± S.E.M.; when stated, n refers to the number of experimental animals. GraphPad Prism 7 software was used for statistical analysis. An unpaired Student’s t test was used for comparisons between control and TNBS-induced rats in the following analyses: Emax and EC_50_ values, dry tissue weight and wet-to-dry, Ang II content of each colonic segment and AT_1_ and AT_2_ expression as well as MPO activity. To evaluate the effect of the antagonists in the response to a single concentration of Ang II, a paired Student’s *t* test was used. Two-way ANOVA was used to look for interactions between two factors. Anywhere, a *p* value lower than 0.05 was considered statistically significant.

## Figures and Tables

**Table 1:** E_max_ (mN/g and %ACh) for Ang II-mediated contraction in the PC, MC and DC of control and TNBS-induced rats.

	PC	MC	DC
	Emax (mN/g)	Emax (mN/g)	Emax (mN/g)
**Control**	199.7 ± 27.38	334.3 ± 61.72	329.8 ± 51.59
**TNBS**	103.9 ± 25.05 *	43.76 ± 12.86 **	27.12 ± 7.295 *
	**Emax (% ACh)**	**Emax (% ACh)**	**Emax (% ACh)**
**Control**	100.2 ± 21.42	115.2 ± 5.009	142.1 ± 18.98
**TNBS**	102.8 ± 29.95	43.11 ± 9.518 *	467.3 ± 80.05 *

* *p* < 0.05 vs. corresponding CTRL animals; ** *p* < 0.0001 vs. corresponding CTRL animals.

**Table 2:** Macroscopic score (MaS).

Macroscopic Score (MaS)		Adhesions to Adjacent Organs	Colon Thickness	Mucosal Edema/ Hyperemia	Mucosal Ulcers
**Score**	**0**	Absent	Normal	Absent	Absent
	**1**	Mild/focal	Mild	Mild	Single
	**2**	Moderate/zonal	Moderate	Moderate	At one site
	**3**	Severe/diffuse	Marked increased	Severe	At more sites

**Table 3:** qPCR conditions for AT_1_ and AT_2_ receptors’ expression analysis.

**Genes of Interest**		
**AT_1_ receptor**	**Forward**	5′-TCTGGATAAATCACACAACCCTC-3′
	**Reverse**	5′-GAGTTGGTCTCAGACACTATTCG-3′
**AT_2_ receptor**	**Forward**	5′-CTGGCAAGCATCTTATGTAGTTC-3′
	**Reverse**	5′-ACAAGCATTCACACCTAAGTATTC-3′
**Housekeeping Genes**		
**Papbn-1**	**Forward**	5′-TATGGTGCGACAGCAGAAGA-3′
	**Reverse**	5′-TATGCAAACCCTTTGGGATG-3′
**Hprt-1**	**Forward**	5′-CCCAGCGTCGTGATTAGTGATG-3′
	**Reverse**	5′-TTCAGTCCTGTCCATAATCAGTCC-3′
**Hmbs**	**Forward**	5′-TCTAGATGGCTCAGATAGCATGCA-3′
	**Reverse**	5′-TGGACCATCTTCTTGCTGAACA-3′

## Data Availability

Data is available upon request.

## References

[1] Ng S.C., Shi H.Y., Hamidi N., E Underwood F., Tang W., I Benchimol E., Panaccione R., Ghosh S., Wu J.C.Y., Chan F.K.L. (2017). Worldwide incidence and prevalence of inflammatory bowel disease in the 21st century: A systematic review of population-based studies. Lancet.

[2] Strohl M., Gonczi L., Kurt Z., Bessissow T., Lakatos P.L. (2018). Quality of care in inflammatory bowel diseases: What is the best way to better outcomes?. World J. Gastroenterol..

[3] Büsch K., Sonnenberg A., Bansback N. (2014). Impact of Inflammatory Bowel Disease on Disability. Curr. Gastroenterol. Rep..

[4] Geremia A., Biancheri P., Allan P., Corazza G.R., Di Sabatino A. (2014). Innate and adaptive immunity in inflammatory bowel disease. Autoimmun. Rev..

[5] Maloy K.J., Powrie F. (2011). Intestinal homeostasis and its breakdown in inflammatory bowel disease. Nat. Cell Biol..

[6] Leitner G.C., Vogelsang H. (2016). Pharmacological- and non-pharmacological therapeutic approaches in inflammatory bowel disease in adults. World J. Gastrointest. Pharmacol. Ther..

[7] Capettini L.S., Montecucco F., Mach F., Stergiopulos N., Santos R., da Silva R.F. (2012). Role of renin-angiotensin system in inflammation, immunity and aging. Curr. Pharm. Des..

[8] Shi Y., Liu T., He L., Dougherty U., Chen L., Adhikari S., Alpert L., Zhou G., Lui W., Wang J. (2016). Activation of the Renin-Angiotensin System Promotes Colitis Development. Sci. Rep..

[9] Liu C., Xiao L., Li F., Zhang H., Li Q., Liu H., Fu S., Li C., Zhang X., Wang J. (2014). Generation of outbred Ace2 knockout mice by RNA transfection of TALENs displaying colitis reminiscent pathophysiology and inflammation. Transgenic Res..

[10] Yisireyili M., Uchida Y., Yamamoto K., Nakayama T., Cheng X.W., Matsushita T., Nakamura S., Murohara T., Takeshita K. (2018). Angiotensin receptor blocker irbesartan reduces stress-induced intestinal inflammation via AT1a signaling and ACE2-dependent mechanism in mice. Brain Behav. Immun..

[11] Zizzo M.G., Caldara G., Bellanca A., Nuzzo D., Di Carlo M., Serio R. (2020). PD123319, angiotensin II type II receptor antagonist, inhibits oxidative stress and inflammation in 2, 4-dinitrobenzene sulfonic acid-induced colitis in rat and ameliorates colonic contractility. Inflammopharmacology.

[12] Garg M., Angus P.W., Burrell L.M., Herath C., Gibson P.R., Lubel J.S. (2012). Review article: The pathophysiological roles of the renin-angiotensin system in the gastrointestinal tract. Aliment. Pharmacol. Ther..

[13] Paul M., Mehr A.P., Kreutz R. (2006). Physiology of Local Renin-Angiotensin Systems. Physiol. Rev..

[14] Ewert S., Spak E., Olbers T., Johnsson E., Edebo A., Fändriks L. (2006). Angiotensin II induced contraction of rat and human small intestinal wall musculature in vitro. Acta Physiol..

[15] Mastropaolo M., Zizzo M.G., Mule F., Serio R. (2013). Angiotensin II contractile effects in mouse colon: Role for pre- and post-junctional AT 1A receptors. Acta Physiol..

[16] Hawcock A.B., Barnes J.C. (1993). Pharmacological characterization of the contractile responses to angiotensin analogues in guinea-pig isolated longitudinal muscle of small intestine. Br. J. Pharmacol..

[17] Zizzo M.G., Auteri M., Amato A., Caldara G., Nuzzo D., Di Carlo M., Serio R. (2017). Angiotensin II type II receptors and colonic dysmotility in 2,4-dinitrofluorobenzenesulfonic acid-induced colitis in rats. Neurogastroenterol. Motil..

[18] Valès S., Touvron M., Van Landeghem L. (2018). Enteric glia: Diversity or plasticity?. Brain Res..

[19] Coelho-Aguiar J.M., Bon-Frauches A.C., Gomes A.L., Verissimo C.P., Aguiar D.P., Matias D., Thomasi B.B., Gomes A.S., Brito G.A., Moura-Neto V. (2015). The enteric glia: Identity and functions. Glia.

[20] Gulbransen B.D., Christofi F.L. (2018). Are We Close to Targeting Enteric Glia in Gastrointestinal Diseases and Motility Disorders?. Gastroenterology.

[21] Nasser Y., Fernandez E., Keenan C.M., Ho W., Oland L.D., Tibbles L.A., Schemann M., Macnaughton W.K., Rühl A., Sharkey K.A. (2006). Role of enteric glia in intestinal physiology: Effects of the gliotoxin fluorocitrate on motor and secretory function. Am. J. Physiol. Liver Physiol..

[22] Pochard C., Coquenlorge S., Freyssinet M., Naveilhan P., Bourreille A., Neunlist M., Rolli-Derkinderen M. (2018). The multiple faces of inflammatory enteric glial cells: Is Crohn’s disease a gliopathy?. Am. J. Physiol. Liver Physiol..

[23] Chow A.K., Gulbransen B.D. (2017). Potential roles of enteric glia in bridging neuroimmune communication in the gut. Am. J. Physiol. Liver Physiol..

[24] Bradley J.S., Parr E.J., Sharkey K.A. (1997). Effects of inflammation on cell proliferation in the myenteric plexus of the guinea-pig ileum. Cell Tissue Res..

[25] Lies B., Beck K., Keppler J., Saur D., Groneberg D., Friebe A. (2015). Nitrergic signalling via interstitial cells of Cajal regulates motor activity in murine colon. J. Physiol..

[26] Schneider S., Wright C.M., Heuckeroth R.O. (2019). Unexpected Roles for the Second Brain: Enteric Nervous System as Master Regulator of Bowel Function. Annu. Rev. Physiol..

[27] Beck K., Friebe A., Voussen B. (2018). Nitrergic signaling via interstitial cells of Cajal and smooth muscle cells influences circular smooth muscle contractility in murine colon. Neurogastroenterol. Motil..

[28] Antonioli L., Colucci R., Pellegrini C., Giustarini G., Tuccori M., Blandizzi C., Fornai M. (2013). The role of purinergic pathways in the pathophysiology of gut diseases: Pharmacological modulation and potential therapeutic applications. Pharmacol. Ther..

[29] Bernardini N., Segnani C., Ippolito C., de Giorgio R., Colucci R., Faussone-Pellegrini M.S., Chiarugi M., Campani D., Castagna M., Mattii L. (2011). Immunohistochemical analysis of myenteric ganglia and interstitial cells of Cajal in ulcerative colitis. J. Cell. Mol. Med..

[30] Rumessen J.J., Vanderwinden J.-M., Horn T. (2010). Crohn’s disease of the colon: Ultrastructural changes in submuscular interstitial cells of Cajal. Cell Tissue Res..

[31] Antoniou E., Margonis G.A., Angelou A., Pikouli A., Argiri P., Karavokyros I., Papalois A., Pikoulis E. (2016). The TNBS-induced colitis animal model: An overview. Ann. Med. Surg..

[32] Tanović A., Fernández E., Jiménez M. (2006). Alterations in intestinal contractility during inflammation are caused by both smooth muscle damage and specific receptor-mediated mechanisms. Croat. Med. J..

[33] Kaschina E., Unger T. (2003). Angiotensin AT1/AT2 receptors: Regulation, signalling and function. Blood Press..

[34] Jaszewski R., Tolia V., Ehrinpreis M.N., Bodzin J.H., Peleman R.R., Korlipara R., Weinstock J.V. (1990). Increased colonic mucosal angiotensin I and II concentrations in Crohn’s colitis. Gastroenterology.

[35] Matsuda T., Suzuki J., Furuya K., Masutani M., Kawakami Y. (2001). Serum angiotensin I-converting enzyme is reduced in Crohn’s disease and ulcerative colitis irrespective of genotype. Am. J. Gastroenterol..

[36] Mastropaolo M., Zizzo M.G., Auteri M., Caldara G., Liotta R., Mule F., Serio R. (2015). Activation of angiotensin II type 1 receptors and contractile activity in human sigmoid colon in vitro. Acta Physiol..

[37] Ferreira Duarte M., Pinto-Rodrigues T., Menezes-Pinto D., Esteves-Monteiro M., Gonçalves-Monteiro S., Capas-Peneda S., Magro F., Dias-Pereira P., Morato M., Duarte-Araújo M. (2021). TNBS-induced colitis in Rattus norgevicus: A categorization proposal. Exp. Anim..

[38] Katada K., Yoshida N., Suzuki T., Okuda T., Mizushima K., Takagi T., Ichikawa H., Naito Y., Cepinskas G., Yoshikawa T. (2008). Dextran sulfate sodium-induced acute colonic inflammation in angiotensin II type 1a receptor deficient mice. Inflamm. Res..

[39] Mizushima T., Sasaki M., Ando T., Wada T., Tanaka M., Okamoto Y., Ebi M., Hirata Y., Murakami K., Mizoshita T. (2010). Blockage of angiotensin II type 1 receptor regulates TNF-alpha-induced MAdCAM-1 expression via inhibition of NF-kappaB translocation to the nucleus and ameliorates colitis. Am. J. Physiol. Gastrointest. Liver Physiol..

[40] Morato M., Pinho D., Sousa T., Guimarães S., Moura D., Albino-Teixeira A. (2006). Pre- and postjunctional effects of angiotensin II in hypertension due to adenosine receptor blockade. Eur. J. Pharmacol..

[41] Balt J.C., Mathy M.-J., Nap A., Pfaffendorf M., Van Zwieten P.A. (2002). Prejunctional and Postjunctional Inhibitory Actions of Eprosartan and Candesartan in the Isolated Rabbit Mesenteric Artery. J. Cardiovasc. Pharmacol..

[42] Moura D., Pinheiro H., Paiva M.Q., Guimaraes S. (1999). Prejunctional effects of angiotensin II and bradykinin in the heart and blood vessels. J. Auton. Pharmacol..

[43] Mota A., Guimarães S. (2002). Interaction between α2-autoreceptors and receptors mediating the effects of angiotensin II and bradykinin in the heart of newborn rats. Eur. J. Pharmacol..

[44] Shetty S.S., Delgrande D. (2000). Differential inhibition of the prejunctional actions of angiotensin II in rat atria by valsartan, irbesartan, eprosartan, and losartan. J. Pharmacol. Exp. Ther..

[45] Cox S.L., Trendelenburg A.U., Starke K. (1999). Prejunctional angiotensin receptors involved in the facilitation of noradrenaline release in mouse tissues. Br. J. Pharmacol..

[46] Rump L., Schuster M., Wilde K., Schollmeyer P. (1990). Modulation of noradrenaline release from rat cortical kidney slices: Effects of angiotensin I and II. Br. J. Clin. Pharmacol..

[47] Furness J.B. (2012). The enteric nervous system and neurogastroenterology. Nat. Rev. Gastroenterol. Hepatol..

[48] Kinoshita K., Horiguchi K., Fujisawa M., Kobirumaki F., Yamato S., Hori M., Ozaki H. (2006). Possible involvement of muscularis resident macrophages in impairment of interstitial cells of Cajal and myenteric nerve systems in rat models of TNBS-induced colitis. Histochem. Cell Biol..

[49] Altdorfer K., Bagameri G., Donath T., Feher E. (2002). Nitric oxide synthase immunoreactivity of interstitial cells of Cajal in experimental colitis. Inflamm. Res..

[50] Vieira C., Ferreirinha F., Magalhães-Cardoso M.T., Silva I., Marques P., Correia-De-Sá P. (2017). Post-inflammatory Ileitis Induces Non-neuronal Purinergic Signaling Adjustments of Cholinergic Neurotransmission in the Myenteric Plexus. Front. Pharmacol..

[51] Jabbur S.J., El-Kak F.H., Nassar C.F. (1988). The enteric nervous system—an overview. Med. Res. Rev..

[52] Shiina T., Gurung Y.B., Suzuki Y., Takewaki T., Shimizu Y. (2013). Alteration of neuromuscular transmissions in the hamster colon following the resolution of TNBS-induced colitis. J. Physiol. Sci..

[53] Sung T.-S., La J.-H., Kim T.-W., Yang I.-S. (2006). Alteration of nitrergic neuromuscular transmission as a result of acute experimental colitis in rat. J. Vet. Sci..

[54] Boulanger C.M., Morrison K.J., Vanhoutte P.M. (1994). Mediation by M3-muscarinic receptors of both endothelium-dependent contraction and relaxation to acetylcholine in the aorta of the spontaneously hypertensive rat. Br. J. Pharmacol..

[55] Tangsucharit P., Takatori S., Zamami Y., Goda M., Pakdeechote P., Kawasaki H., Takayama F. (2016). Muscarinic acetylcholine receptor M1 and M3 subtypes mediate acetylcholine-induced endothelium-independent vasodilatation in rat mesenteric arteries. J. Pharmacol. Sci..

[56] Anderson J.C.D., Kendig D.M., Al-Qudah M., Mahavadi S., Murthy K.S., Grider J.R. (2014). Role of various kinases in muscarinic M3 receptor-mediated contraction of longitudinal muscle of rat colon. J. Smooth Muscle Res..

[57] Barnes J., Costall B., Horovitz Z., Naylor R. (1989). Angiotensin II inhibits the release of [3H]acetylcholine from rat entorhinal cortex in vitro. Brain Res..

[58] Harrington A.M., Peck C.J., Liu L., Burcher E., Hutson J.M., Southwell B.R. (2010). Localization of muscarinic receptors M1R, M2R and M3R in the human colon. Neurogastroenterol. Motil..

[59] Kodama Y., Iino S., Shigemasa Y., Suzuki H. (2010). Properties of acetylcholine-induced relaxation of smooth muscle isolated from the proximal colon of the guinea-pig. J. Smooth Muscle Res..

[60] Porter A.J., A Wattchow D., Brookes S.J.H., Costa M. (2002). Cholinergic and nitrergic interneurones in the myenteric plexus of the human colon. Gut.

[61] Kortezova N., Shikova L., Papasova M. (1998). Participation of M1 receptors in NO pathway in cat ileum. Acta Physiol. Pharmacol. Bulg..

[62] Li M., Johnson C.P., Adams M.B., Sarna S.K. (2002). Cholinergic and nitrergic regulation of in vivo giant migrating contractions in rat colon. Am. J. Physiol. Liver Physiol..

[63] Rychlik A., Gonkowski S., Nowicki M., Calka J. (2017). Inflammatory bowel disease affects density of nitrergic nerve fibers in the mucosal layer of the canine gastrointestinal tract. Can. J. Vet. Res..

[64] Green C.L., Ho W., Sharkey K.A., McKay D.M. (2004). Dextran sodium sulfate-induced colitis reveals nicotinic modulation of ion transport via iNOS-derived NO. Am. J. Physiol. Gastrointest. Liver Physiol..

[65] Lundberg S., Holst M., Hellström P.M. (2006). Expression of iNOS mRNA associated with suppression of colonic contraction in rat colitis. Acta Physiol..

[66] Morris G.P., Beck P.L., Herridge M.S., Depew W.T., Szewczuk M.R., Wallace J.L. (1989). Hapten-induced model of chronic inflammation and ulceration in the rat colon. Gastroenterology.

[67] Wirtz S., Popp V., Kindermann M., Gerlach K., Weigmann B., Fichtner-Feigl S., Neurath M.F. (2017). Chemically induced mouse models of acute and chronic intestinal inflammation. Nat. Protoc..

[68] Dinis-Oliveira R., Remião F., Duarte J., Ferreira R., Navarro A.S., Bastos M., Carvalho F. (2006). P-glycoprotein induction: An antidotal pathway for paraquat-induced lung toxicity. Free Radic. Biol. Med..

[69] Lowry O., Rosebrough N., Farr A.L., Randall R. (1951). Protein Measurement with the Folin Phenol Reagent. J. Biol. Chem..

[70] Marquez L.A., Dunford H.B. (1997). Mechanism of the Oxidation of 3,5,3‘,5‘-Tetramethylbenzidine by Myeloperoxidase Determined by Transient- and Steady-State Kinetics. Biochemistry.

[71] Chong H.L., Ruben P.C. (2008). Interaction between Voltage-Gated Sodium Channels and the Neurotoxin, Tetrodotoxin. Channels.

[72] Paulsen R.E., Contestabile A., Villani L., Fonnum F. (1987). An In Vivo Model for Studying Function of Brain Tissue Temporarily Devoid of Glial Cell Metabolism: The Use of Fluorocitrate. J. Neurochem..

[73] Kraus R.L., Li Y., Gregan Y., Gotter A.L., Uebele V.N., Fox S.V., Doran S.M., Barrow J.C., Yang Z.-Q., Reger T.S. (2010). In Vitro Characterization of T-Type Calcium Channel Antagonist TTA-A2 and In Vivo Effects on Arousal in Mice. J. Pharmacol. Exp. Ther..

[74] Sabatino L., Costagli C., Lapi D., Del Seppia C., Federighi G., Balzan S., Colantuoni A., Iervasi G., Scuri R. (2018). Renin-Angiotensin System Responds to Prolonged Hypotensive Effect Induced by Mandibular Extension in Spontaneously Hypertensive Rats. Front. Physiol..

[75] Livak K.J., Schmittgen T.D. (2001). Analysis of relative gene expression data using real-time quantitative PCR and the 2(-Delta Delta C(T)) Method. Methods.

